# GroEL Secreted from *Bacillus subtilis* Natto Exerted a Crucial Role for Anti-Inflammatory IL-10 Induction in THP-1 Cells

**DOI:** 10.3390/microorganisms11051281

**Published:** 2023-05-14

**Authors:** Taisuke Uesugi, Suguru Mori, Kazuhiko Miyanaga, Naoyuki Yamamoto

**Affiliations:** 1 School of Life Science and Technology, Tokyo Institute of Technology, Yokohama 226-8501, Kanagawa, Japan; 2 Ezaki Glico Co., Ltd., 4-6-5 Utajima, Nishiyodogawa-ku, Osaka 555-8502, Osaka, Japan; 3 Department of Infection and Immunity, School of Medicine, Jichi Medical University, 3311-1, Yakushiji, Shimotsuke-Shi 329-0498, Tochigi, Japan

**Keywords:** *Bacillus subtilis* natto, immunomodulatory effect, THP-1 DC, IL-10, GroEL, chaperon protein

## Abstract

Although diverse immunomodulatory reactions of probiotic bacteria have been reported, this effect via *Bacillus subtilis* natto remains unclear, despite its long consumption history in Japan and usage in Natto production. Hence, we performed a comparative analysis of the immunomodulatory activities of 23 types of *B. subtilis* natto isolated from Natto products to elucidate the key active components. Among the isolated 23 strains, the supernatant from *B. subtilis* strain 1 fermented medium showed the highest induction of anti-inflammatory IL-10 and pro-inflammatory IL-12 in THP-1 dendritic cells (THP-1 DC) after co-incubation. We isolated the active component from strain 1 cultured medium and employed DEAE-Sepharose chromatography with 0.5 M NaCl elution for fractionation. IL-10-inducing activity was specific to an approximately 60 kDa protein, GroEL, which was identified as a chaperone protein and was significantly reduced with anti-GroEL antibody. Differential expression analysis of strains 1 and 15, which had the lowest cytokine-producing activity, showed a higher expression of various genes involved in chaperones and sporulation in strain 1. Furthermore, GroEL production was induced in spore-forming medium. The present study is the first to show that the chaperone protein GroEL, secreted by *B. subtilis* natto during sporulation, plays a crucial role in IL-10 and IL-12 production in THP-1 DC.

## 1. Introduction

Probiotics are often used to maintain the intestinal microbial balance, control gastrointestinal disorders, induce host immunomodulation, and prevent pathogenic bacterial infection [1,2,3,4]. Lactic acid bacteria (LAB) are promising probiotics that play a pivotal role in the production of functional food components with health benefits for humans and animals. Therefore, useful LAB have been isolated from traditional fermented food products, human faecal samples, and environmental niches [5], including fruits and vegetables, for use in dairy products. The host immune function of the intestinal microbiota is elucidated via their interactions with the gut immune system [6]. The immunomodulatory effects of lactobacilli are closely linked to their uptake into the gut-associated lymphoid tissue and the modulation of mucosal immune responses. Probiotic lactobacilli are strong inducers of pro-inflammatory cytokines such as interleukin (IL)-12 and tumour necrosis factor (TNF)-α in the gut [7]. The stimulation of the host immune function by probiotic lactobacilli is explained by the initiation of bacterial interactions with the gut immune system [7]. Previous studies have demonstrated various host immune responses via host-bacterial interactions or host-postbiotics (bacterial metabolites) involving conserved bacterial ligands and pattern recognition receptors (PRRs), such as Toll-like receptors (TLRs), on dendritic cells (DCs) [8]. Microbial-associated molecular patterns (MAMPs) modulate multiple host immune systems. Lipopolysaccharides (LPSs); lipoproteins; peptidoglycans; polysaccharide A; lipoteichoic acid (LTA); microbial RNA, DNA, and CpG motifs have been reported to be MAMP components [9]. Crosstalk between MAMPs in lactobacilli and host PRRs modulates the signalling of certain pathways after interaction with TLR, nuclear factor-kappa B (NF-κB), and mitogen-activated protein kinase signalling pathways [9].

*Bacillus subtilis*, a Gram-positive spore-forming bacterium living in natural environments, can produce proteases to degrade proteins for utilisation of the released peptides as nitrogen sources. *B. subtilis* natto (*B. subtilis* var. natto) [10], which can produce sticky materials composed of poly gamma glutamic acid (γPGA), fructan, and specific flavour, is used for the production of the traditional soybean fermentation product “Natto”, a long-standing Japanese superfood [11]. *B. subtilis* natto is considered to have probiotic potential with health benefits for humans and as a feed additive for animals [12]. Natto-containing *B. subtilis* natto is known for its multiple probiotic functions [13], such as the control of intestinal microbiota and immunomodulatory effect [14,15,16,17]. The highest quartile of natto intake was also significantly associated with a decreased risk of mortality due to ischemic stroke [18]. Additionally, the anti-inflammatory effect of *B. subtilis* acts on host immune cell populations to stimulate peripheral blood mononuclear cells [19]. Spore-forming *B. subtilis* cells significantly increase the production of interleukin IL-12 protein [20] whereby γPGA activates to produce IFNγ and TNFα, as well as IL-12 [21]. However, the immunomodulatory effects of the *B. subtilis* natto species and its contributions remain unknown.

In this study, via comparative analysis, we investigated the immunomodulatory properties of *B. subtilis* natto, which is used in various natural products in Japan. Furthermore, we aimed to purify and identify the active component of the most active *B. subtilis* natto strain. Additionally, we elucidated the mechanism of action of the purified component in THP-1 DCs based on the differential gene expression analysis to highlight the immunomodulatory activity of *B. subtilis* natto.

## 2. Materials and Methods

### 2.1. Bacterial Strains and Fermentation

All *B. subtilis* natto strains isolated from natto products were listed in Table 1 and were cultured in 10 mL of trypticase soy (TS) broth for 20 h at 37 °C after inoculation of 0.5 mL of preculture. The cells were then harvested by centrifugation at 10,000× *g* for 5 min and washed with 10 mL of phosphate buffered saline (PBS) (137 mM NaCl, 10 mM Na_2_HPO_4_, 2.7 mM KCl, and 1.8 mM KH_2_PO_4_, pH 7.2). *Lactobacillus acidophilus* JCM 1132 was obtained from the Japan Collection of Microorganisms (JCM) and cultured in MRS (de Man-Rogosa-Sharpe) medium (Becton, Dickinson and Company, MD, USA) for 20 h at 37 °C. The cells were harvested by centrifugation at 10,000× *g* for 5 min and washed with 10 mL of PBS.

### 2.2. Cytokine Measurement

Human THP-1 monocytes were obtained from RIKEN Cell Bank (JRCB0112). THP-1 cells were cultured in Roswell Park Memorial Institute 1640 (RPMI 1640) supplemented with 10% fetal bovine serum (FBS), 100 U/mL penicillin and 100 µg/mL streptomycin at 37 °C in a 5% CO_2_ humidified incubator. THP-1 cells were differentiated in 96-well culture plates at 5.0 × 10^4^ cells per well by incubation with 100 nM phorbol 2-myristate 13-acetate (PMA; Adipogen Life Science, Liestal, Switzerland) for 3 days to measure cytokine production in THP-1 DC. Then, the PBS-washed *B. subtilis* natto cells or Natto product were added to THP-1 DC (MOI = 10) at 5.0 × 10^5^ cells per well, and IL-4 (100 ng/mL) was added to IL-10 or IFN-γ (100 ng/mL) for IL-12 p40 production. The cultured medium supernatant or the supernatant from the Natto product was obtained from an equal number of cells, or in the case of fractionated samples, 10 µL of each sample were added to THP-1 DC. After incubation at 37 °C under 5% CO_2_ for 24 h, the supernatant containing cytokines was collected and measured using an enzyme-linked immunosorbent assay (ELISA) kit. The IL-10 ELISA kit was obtained from BioLegend Inc. (San Diego, CA, USA), and the IL-12 p40 ELISA kit was purchased from R&D Systems (Minneapolis, MN, USA). Fluorescence was measured in triplicates using a plate reader (Varioskan LUX SkanIt Software 4.0; Thermo Fisher Scientific). For the inhibitory effect on IL-10-inducing activity, an anti-GroEL antibody (Enzo Life Sciences Inc., Farmingdale, NY, USA) was added to THP-1 DC with strain 1 supernatant. For receptor analysis, an anti-TLR2 antibody (Sino Biological Inc., Peking, China) or anti-TLR4 antibody (Sino Biological Inc., Beijing, China) was pre-incubated with THP-1 DC prior to the addition of purified GroEL. Thereafter, 10 µL of strain 1 supernatant or purified GroEL was added to THP-1 DC and incubated for 24 h prior to IL-10 measurement.

### 2.3. Purification of the Immunomodulatory Component

*B. subtilis* natto strain 1 was cultured in 100 mL of trypticase soy (TS) broth for 18 h at 37 °C, and the cells were harvested by centrifugation at 10,000× *g* for 5 min, then washed with 10 mL of PBS. The washed cells were resuspended in 100 mL PBS, and the suspension was heat-treated at 100 °C for 10 min. For the isolation of cell surface proteins, the washed cells were suspended in 100 mL of 5 M LiCl and incubated for 30 min at 37 °C. Then, the supernatants were collected by centrifugation at 10,000× *g* for 5 min and dialysed against 50 times the volume of PBS. The supernatant was filtered with a 0.2 µm sterile syringe filter, and 100 mL of the supernatant was applied onto a column (Φ12 mm × 25 cm) filled with 5 mL of DEAE-Sepharose FastFlow (Sigma-Aldrich, St. Louis, MO, USA) previously equilibrated with 10 mM Tris-Cl pH 7.5 (Tris-buffer). The column was washed with 15 mL of Tris-buffer before protein elution with Tris-buffer containing 0, 50, 100, 150, 200, 300, or 500 mM NaCl. Each 15 mL fraction was concentrated to 1 mL using an Amicon Ultra-4 Centrifugal Filter Unit. The immunomodulatory activity and protein levels of the encapsulated samples were measured. GroEL was purified by affinity column chromatography. To prepare the affinity resin, an anti-GroEL antibody (Enzo Life Sciences Inc., Farmingdale, NY, USA) was dialysed against NaHCO_2_ pH 8.5 and covalently coupled to the affinity resin. About 100 µg of antibody was mixed with 1 mL of Profinity Epoxide (Bio-Rad Laboratories Inc., Hercules, CA, USA) to couple with the resin. The active fraction eluted from DEAE-Sepharose was loaded onto an affinity column pre-equilibrated with PBS. After washing with 10 mL of PBS, the bound proteins were eluted from the column by washing with 3 M KSCN. The eluted samples were dialysed against tris-buffered saline.

### 2.4. Sporulation of B. subtilis Natto Strains

*B. subtilis* natto strain 1 was sporulated by cultivation in Difco Sporulation Medium (DSM) for 48 h at 37 °C as described previously [10]. The spore-formed cells were centrifuged at 10,000× *g* for 5 min, and the supernatant was filtered with a 0.2 µm sterile syringe filter prior to measurement of the immunomodulatory activity. To measure the spore-forming rate, culture suspensions were heated in a water bath for 30 min at 65 °C (spore cells) or without heating (total cells). Subsequently, the number of viable cells in each sample was measured using trypticase soy agar for 18 h at 37 °C.

### 2.5. SDS-PAGE, Western Blotting, and Protein Identification

Proteins in each fraction from the DEAE-Sepharose and affinity chromatography were analysed using 10% SDS-PAGE. The proteins were mixed with sample buffer (×6: 125 nM Tris-HCl, 4% SDS, 20% glycerol, 0.012% bromophenol blue, and 10% 2-mercaptoethanol), heated for 5 min at 95 °C, and analysed by SDS-PAGE, according to the method of Laemmli [22]. The protein bands were visualised by staining the gel with CBB. Protein Ladder One Plus (Nacalai Tesque) was used as a size marker. Proteomic analysis was performed to identify the proteins. Protein bands were excised from the SDS-PAGE gels after CBB staining. To remove the dye from the gel, a destaining solution (30% acetonitrile and 50 mM NH_4_HCO_3_) was added and the gel was incubated for 30 min. Subsequently, 60% acetonitrile and 20 mM NH_4_HCO_3_ were added to remove water from the gel. Next, 5% (*w*/*w*) of trypsin (Promega, Japan, Tokyo) was added to the dried gel and incubated at 37 °C for 12 h. The peptides released from the gel were analysed by mass spectrometry using an UltrafleXtreme TOF/TOF MS (Bruker Daltonics GmbH, Bremen, Germany) operating in the positive reflection ion mode between *m*/*z* 0 and 5000 Da. For Western blot analysis, proteins released from 1.0 × 10^10^ cells by washing with 5M LiCl were concentrated to 50 μL by using an Amicon Ultra-4 Centrifugal Filter Unit and analysed by SDS-PAGE. Then, the proteins in the gel were transferred to a polyvinylidene fluoride membrane (Wako Pure Chemical Industries, Ltd., Osaka, Japan) in Tris-glycine buffer at pH 8.3 (190 mM glycine, 5 mM Tris-Cl, and 20% methanol) for 1 h at 200 mA. Proteins were detected using an anti-GroEL antibody (Enzo Life Sciences, Inc., Farmingdale, NY, USA). Then, the GroEL-specific band was visualized by adding of 2 mL of Chemi-Lumi One (Nakalai, Kyoto, Japan) according to the product manual.

### 2.6. Gene Expression Analysis for B. subtilis Natto 1 and 15

*B. subtilis* natto strains 1 and 15 were cultured for 16 h at 37 °C and cells harvested via centrifugation at 10,000× *g* for 5 min followed by PBS wash. Total RNA extraction employed the RNeasy Mini Kit (QIAGEN, Hilden, Germany). After evaluating the quality and quantity of RNA using a NanoDrop 2000 (Thermo Fisher Scientific, Waltham, MA, USA), equal amounts of RNA samples extracted from triplicate samples of each group were mixed, followed by sequencing of two mixed groups from the triplicate samples: *B. subtilis* natto strain 1 treated group and *B. subtilis* natto strain 15 treated group. We conducted THP-1 DC RNA sequencing at the Bioengineering Lab. Co., Ltd. (Sagamihara, Japan) and analysed differential expression using DESeq (ver. 1.18.0). Corresponding to those in the control group, genes with an adjusted (fold-change) >1.4 were selected for network analysis. Molecular interactions between the upregulated genes were visualised using STRING analysis (https://string-db.org/: accessed on 18 September 2022).

### 2.7. Gene Expression Analysis for THP-1 DC Treated with GroEL

THP-1 cells were seeded at 5.0 × 10^4^ cells per well in a 96-well culture plate and differentiated as described in Section 2.2. Briefly, THP-1 cells were cultured in 96-well plates at 5.0 × 10^4^ cells per well by incubation with 100 nM phorbol 2-myristate 13-acetate for 3 days. Then, each well of THP-1 DCs was supplemented with 10 µL of purified GroEL fraction or PBS and incubated for 4 h at 37 °C under 5% CO_2_. The cells were washed with PBS, and total RNA was extracted. RNA sequencing was performed for two mixed groups from triplicate samples under three iterations: purified GroEL-treated and untreated groups, as described in Section 2.5. Compared to the control group, differentially expressed genes (DEGs) were selected using the criteria 1.4 < fold change and fold-change < 0.5, *p* < 0.05. Genes with an adjusted (fold-change) >1.4 were selected for network analysis. Molecular interactions between upregulated genes were visualised using STRING analysis.

### 2.8. Statistical Analysis

Statistical significance was analysed using GraphPad Prism software package version 9.1. *p*-values less than 0.05 were considered statistically significant.

## 3. Results

### 3.1. Isolation of Bacillus subtilis Natto from Natto Products

All Natto products obtained in the Japanese market are sticky when stirred; this is a well-known characteristic feature of Natto products. The bacteria in each Natto product formed orderly bacterial colonies on the TS agar plates, whereas the Sugoi S-903 product contained two types of colonies. All 23 isolated bacteria were sequenced for the amplified PCR products with specific primers for the V3 and V4 regions of 16S rRNA gene and were predicted to be *B. subtilis*, as listed in Table 1.

### 3.2. Cytokine Inducing Activity

**Figure 1:**
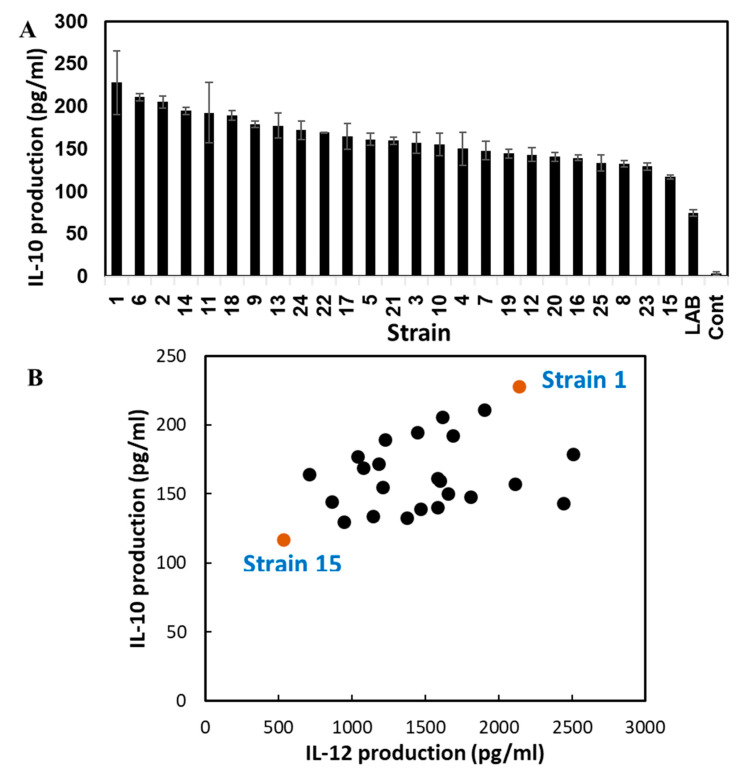
Cytokine-inducing activity of various *B. subtilis* natto strains. IL-10 production in THP-1 DC (**A**) and 2D-plots of IL-10 and IL-12 productions (**B**). “LAB” and “Control” indicate *L. acidophilus* JCM 1132 and medium, respectively.

To understand the cytokine-inducing activities of the 23 isolated types of *B. subtilis* natto strains, the pro-inflammatory cytokine IL-12 and anti-inflammatory cytokine IL-10 from differentiated THP-1 cells were measured. All *B. subtilis* natto strains showed high levels of IL-10- and IL-12-inducing activities (Figure 1A,B). Both the activities differed slightly depending on the tested strain; however, the cytokine-inducing activities of IL-10 and IL-12 were almost parallel (Figure 1B). Among the 23 tested strains, strain 1 showed the highest induction of both IL-10 and IL-12 production, whereas 15 showed the lowest activity for both IL-10 and IL-12 (Figure 1B). Therefore, strain 1 was selected for purification and identification of the active component, and strain 15 was selected as the negative control.

### 3.3. Purification of the Active Components

**Figure 2:**
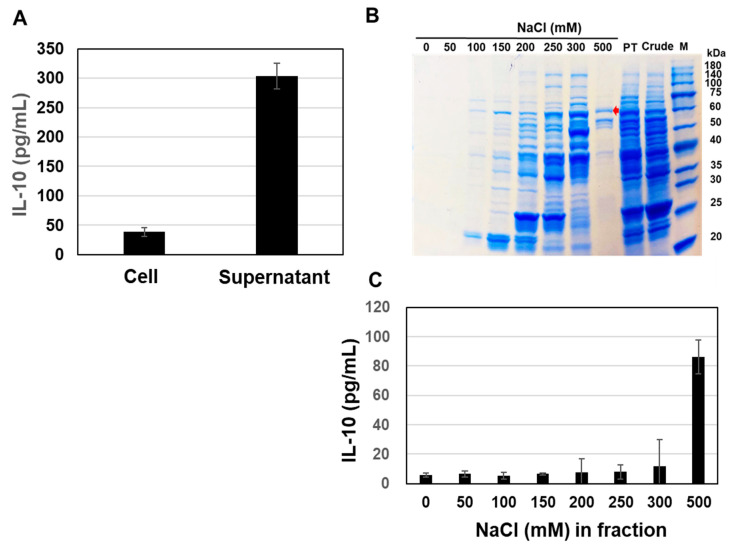
Identification of anti-inflammatory molecules in the supernatant (**A**). Protein expression profile in supernatant from strain 1 and strain 15 (**B**). Fractionation of supernatant from strain 1 with DEAE-Sepharose (**C**). Comparison of IL-10 production in each fraction from strain 1 supernatant. Red arrow shows the specific band in the fraction.

**Figure 3:**
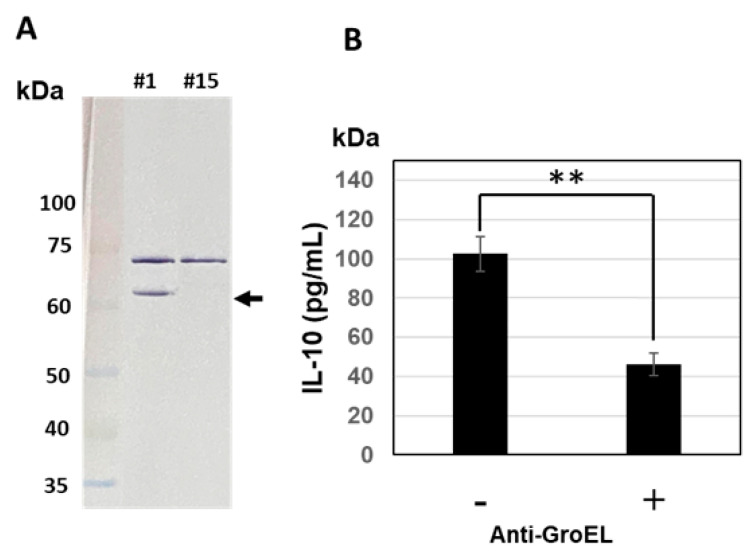
Western blotting analysis for *B. subtilis* strains 1 and 15 with anti-GroEL antibody (**A**) and inhibitory effect of IL-10-inducing activity of strain 1 supernatant by adding of anti-GroEL antibody (**B**). Arrow indicates specific band for GroEL. Significant difference between—antibody group and + antibody group. ** *p* < 0.01 (n = 3).

To confirm the presence of active components in the *B. subtilis* natto culture medium, we compared the activity of the supernatant of strain 1 fermented in TS medium and the surface layer proteins (Slps) released from the cell surface with 5 M LiCl in equal amounts of the cultured medium. IL-10-inducing activity in the supernatant of 1 culture medium (305 pg/mL) was 7.5 times higher than that in the 5M LiCl fraction (40 pg/mL) (Figure 2A). Therefore, strain 1 culture supernatant was selected for purification of the active component and fractionated with 5 mL of DEAE-Sepharose pre-equilibrated with 10 mM Tris-HCl, pH 7.5. As shown in Figure 2B, IL-10-inducing activity was significantly higher with 500 mM NaCl and Tris-Cl (Figure 2C). SDS-PAGE analysis revealed the presence of proteins in the 500 mM NaCl fraction (Figure 3B). We show here an approximately 60 kDa band, indicated by the red arrow, that was specific to the 500 mM fraction. The corresponding bands were extracted from the gel after SDS-PAGE, followed by trypsin digestion as described in the Section 2, prior to proteome analysis. Consequently, the 60 kDa protein was predicted to be GroEL, a chaperone protein that helps hold specific proteins intracellularly and interacts with mucin extracellularly after secretion [23].

### 3.4. Western Blotting Analysis and Inhibition of IL-10-Inducing Activity

Western blot analysis on the culture supernatants with an anti-GroEL antibody verified the heightened level of GroEL in strain 1 compared with strain 15 whereby Figure 3A shows the presence of a unique band in the supernatant of strain 1 but not in that of strain 15, at approximately 60 kDa, corresponding to GroEL. The band observed at approximately 72 kDa in both samples was attributed to non-specific antibody reactions. The GroEL-specific reaction observed at approximately 60 kDa was consistent with the IL-10-inducing activities of supernatant samples from strains 1 and 15, while preincubation with anti-GroEL antibody significantly reduced the IL-10-inducing activity of the strain 1 fermented medium supernatant (Figure 3B). These results suggest that GroEL fractionated from *B. subtilis* natto strain 1 in the fermented medium might be the main contributor to IL-10 induction in THP-1 DC.

### 3.5. Prediction of TLRs for GroEL Interaction

**Figure 4:**
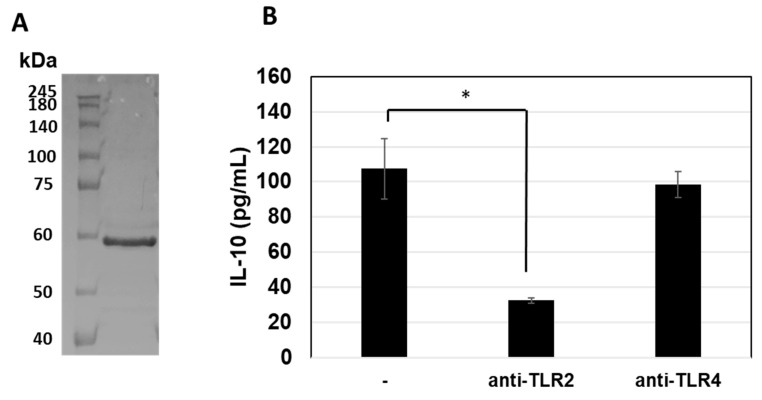
SDS-PAGE of the affinity-purified GroEL (**A**). Reduction of IL-10-inducing activity of GroEL by the treatments with anti-TLR2 and -TLR4 antibodies (**B**). Significant difference from the control (-), * *p* < 0.05 (n = 3).

To verify the expected TLRs for GroEL on THP-1 DC, GroEL was purified from *B. subtilis* natto strain 1 via anti-GroEL antibody coupled affinity resin. An approximately 60 kDa protein was purified from the affinity resin column with 3M KSCN elution (Figure 4A). Thereafter, we evaluated the interactions of the purified GroEL with TLRs on dendritic cells, which is considered a crucial step in host immunomodulatory responses [24]. On top of regulating TLR signalling, several types of TLRs are expressed on the cell surface, such as TLR2 and TIL4, as well as TLR3 and TLR7-9 in intracellular endosomes [24,25]. The expected GroEL receptor on the surface of THP-1 DC was confirmed by measuring IL-10 production using purified GroEL with or without the addition of anti-TLR2 and anti-TLR4 antibodies. As shown in Figure 4, incubation with the anti-TLR2 antibody significantly reduced IL-10, whereas no reduction was observed with the anti-TLR4 antibody. This suggests that interaction of GroEL with TLR2 and TLR2 signalling may activate IL-10 production.

### 3.6. Differentially Expressed Genes in THP-1 DCs after GroEL Treatment

**Figure 5:**
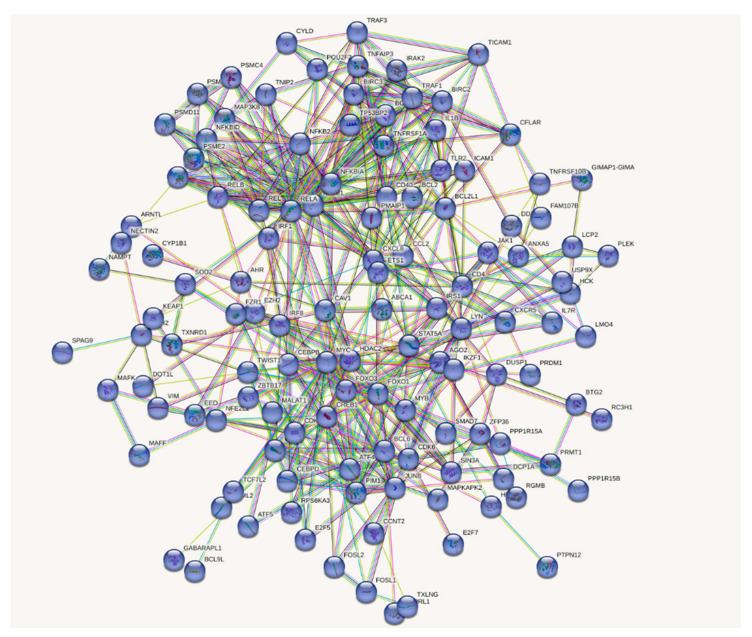
Network analysis of the upregulated gene expressions in THP-1 DC by GroEL treatment.

**Figure 6:**
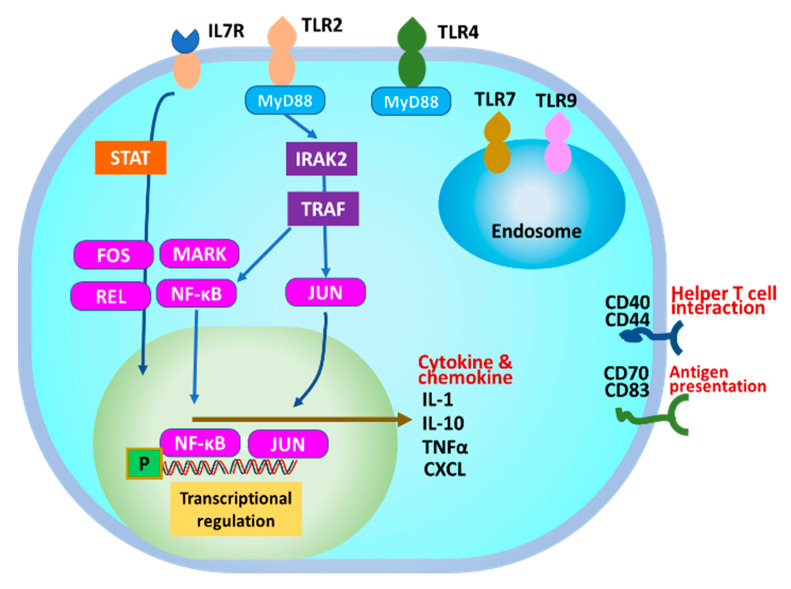
Predicted immunomodulatory effect of GroEL in THP-1 DC. After interactions with a specific receptor, TLR2, STAT, and IRAK2 pathways are activated. Then, transcriptional regulators, JUN, and NF-κB are activated. Finally, specific gene expressions such as cytokines and chemokines are induced.

Here, we verified the IL-10-inducing activity of affinity-purified GroEL and its inhibition by the addition of an anti-GroEL antibody (Figure 4). Thereafter, we performed RNA sequencing of THP-1 DCs to determine the differences in gene expression between purified GroEL (n = 3) and PBS (control, n = 3) to elucidate the mechanism of action underlying the immunomodulatory effect of GroEL produced in *B. subtilis* natto strain 1. To screen DEGs, we juxtaposed the expression profiles of THP-1 DCs treated with GroEL and the control groups. As a result, 763 genes in the GroEL group were upregulated and 1375 genes were downregulated (data not shown) compared to those in the control group whereby DEGs were selected using the criteria 1.4 < fold change and fold change < 0.5, *p* < 0.05, respectively. Molecular interactions between the upregulated genes were visualised using STRING (https://string-db.org/, accessed on 18 September 2022) (available by request). Among the five predicted groups of networks (available by request), the main immunomodulatory responses were detected in the five major clusters. The possible interacting genes suggested to be involved in immune function (Cluster 5) are summarised in Figure 5. The interaction of GroEL with TLR2 on THP-DC may induce signalling pathways, such as IRAK and TRAF; in turn, several transcriptional regulators, such as FOS, MARK, REL, NFKB, and JUN, induce the transcriptions of IL-1, IL-10, TNFa, and several chemokines as illustrated in Figure 6.

### 3.7. Induced Gene Expressions in B. subtilis natto 1

Among the 23 tested kinds of *B. subtilis* natto strains, there were disparities in IL-12 and IL-10 productions; strain 1 showed the highest activity, while strain 15 showed the lowest activity (Figure 1B). We performed RNA sequencing of *B. subtilis* strain 1 (n = 3) and strain 15 (n = 3) to clarify these variations in both strains in terms of gene expression. As a result, strain 1 showed heightened expression in 476 genes compared with those in strain 15 (Table S4) when DEGs selection criteria were 1.4 <fold change of (1/15), *p* < 0.05. Additionally, the expression of most genes involved in sporulation was significantly higher in strain 1 than in strain 15. The molecular interactions among the upregulated genes were visualised using STRING (https://string-db.org/, accessed on 18 September 2022) (Available by request). GroEL gene expression was 1.78-fold higher in strain 1 than that in strain 15. 

**Figure 7:**
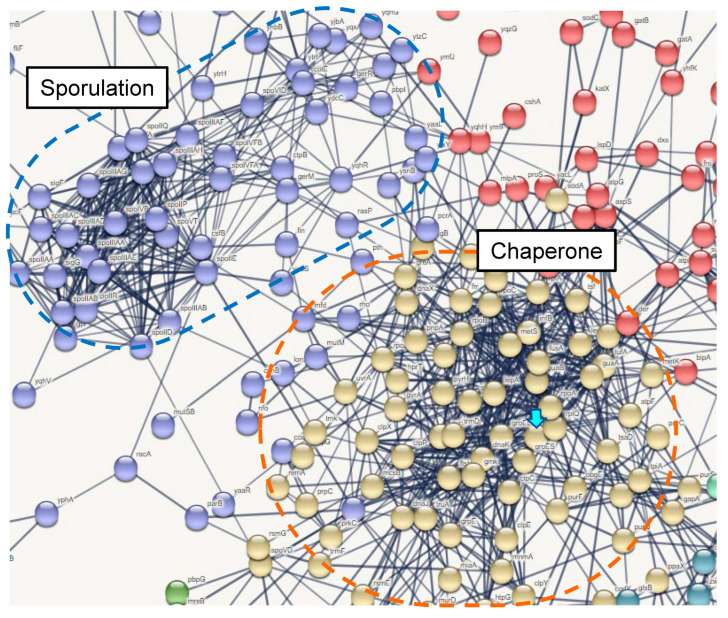
Network analysis for the upregulated genes in *B. subtilis* natto strain 1 compared to *B. subtilis* natto strain 15. Among whole networks, genes involved in Sporulation and Chaperone were showed by blue and red circles. GroEL was indicated by an arrow.

Among the five predicted groups of networks, the main networks involved in Cluster 1 “chaperone” and in Cluster 2 “sporulation” (available by request) are shown in Figure 7. This induction in strain 1 demonstrated the possible interactions of chaperone protein genes such as *GroEL*, *GroES*, *DnaK*, and *DnaJ*; genes for DNA replication, including *DnaX*, *DnaN*, *GyrA*, and *GyrB*; as well as genes involved in sporulation, such as *SpoIIQ*, *SpoIVA*, *SpoIIIAH*, *SpoIIR*, *SpoVID*, *SpoIIR*, *SpoIIP*, *SpoIIABI*, and *SpoIID*.

### 3.8. GroEL Secretion in Spored Cells

**Figure 8:**
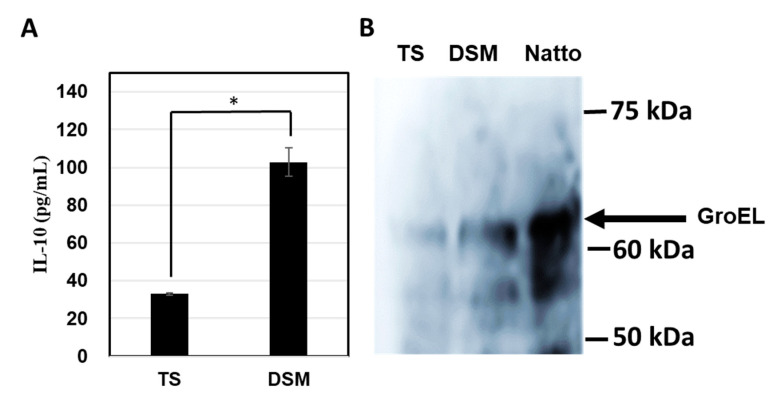
IL-10-inducing activity in THP-1 DC (**A**) and relative GroEL productions in strain 1 cells surface cultured in TS and DSM medium (**B**). Significant difference between TS and DSM, * *p* < 0.05.

Numerous genes involved in sporulation were upregulated in *B. subtilis* 1. Therefore, we cultured strain 1 in DSM to induce sporulation and evaluated their IL-10-inducing activity as well as GroEL production against the TS medium. Ninety-two percent of strain 1 sporulated when it was cultured in DSM medium but did not sporulate in TS medium (3%). As shown in Figure 8A, the IL-10-inducing activity in THP-1 DC increased significantly (3.2-fold) when strain 1 cells were cultured in DSM compared to those cultured in TS medium. Furthermore, to assess the amount of GroEL prepared with strain 1 from the supernatants of TS and DSM, and the suspension of Natto product prepared with strain 1 in PBS, Western blotting analysis was conducted with an anti-GroEL antibody. As shown in Figure 8B, GroEL production in the culture supernatant was significantly higher in DSM than in TS. Interestingly, the amount of GroEL produced from Natto was the highest among the tested samples. These results strongly suggest that GroEL secretion in *B. subtilis* may be induced during sporulation and may contribute to cytokine production.

## 4. Discussion

Previous studies have reported various MAMP components, such as LPSs, lipoproteins, peptidoglycans, polysaccharides, LTA, microbial RNA, DNA, and CpG motifs, that modulate multiple host immune systems [9]. However, the immunomodulatory components of probiotic strains, based on studies with specific species, strains, and different dosages, are not sufficient to understand the key active components and main mechanisms involved. Furthermore, Natto prepared via fermentation with *B. subtilis* natto is considered to have health benefits; however, there is insufficient biological evidence to explain these advantages. Therefore, in the present study, we attempted to identify the key immunomodulatory components of *B. subtilis* natto through a comparative study of 23 commercially available strains and whole-cell extracellular components. Notably, many strains showed higher induction of IL-10 and IL-12 than *L. acidophilus* which has been reported to have strong cytokine-inducing activity [26,27]. Principally, the active component purified from *B. subtilis* natto strain 1 culture medium was a chaperone protein, GroEL, which has not been reported as a general immunomodulatory component in probiotic strains, such as LPSs, lipoproteins, peptidoglycans, polysaccharides, and LTA (reviewed in [28]). In bacilli, the anti-inflammatory effects and molecular mechanisms of exopolysaccharide (EPS) have been reported in *B. amyloliquefaciens* amy-1 [29]. EPS evidently reduces pro-inflammatory factors, phagocytic activity, and oxidative stress in LPS-stimulated THP-1 cells. EPS also ameliorates ear inflammation of mice [29]. Moreover, spore-forming *B. anthracis* induces host immune responses by producing high amounts of RNA present in the spore surface layer for interaction with TLR7 and TLR13 (mice and humans, respectively). In contrast, the GroEL purified in the present study, without EPS or RNA, showed high IL-10-inducing activity. The justification for previously reported active components was likely due to the concentration of the active fraction, without a comparative study with the positive strain. Moreover, gene expression analysis with GroEL showed increased NF-κB (available by rewuest) but induced JNK, which was reported in a previous study in *B. subtilis*. Therefore, this is the first report to demonstrate the immunomodulatory effect of GroEL in *B. subtilis* natto strain which is used in Natto, and the advantage of enriched GroEL in spore-formed Natto.

GroEL derived from *Limosilactobacillus reuteri* inhibited pro-inflammatory cytokines (TNFα, IL-1β, and IFNγ) and repressed haemorrhagic colitis induced in DSS model mice [30]. It also increased anti-inflammatory IL-10 and IL-13 and decreased inflammatory cytokines, such as IFNγ, in the intestine. Mice that were administered recombinant *Streptococcus pneumoniae* GroEL showed a protective effect against challenged *S. pneumoniae* intranasally [31]. These results support our finding that GroEL isolated from *B. subtilis* strain 1 shows immunomodulatory effects on THP-1 DC and may reduce inflammation in animal models. In contrast, GroEL-stimulated NF-κB transcriptional activity was significantly inhibited by anti-TLR2 and anti-TLR4 antibodies. *Porphyromonas gingivalis* GroEL induces its intracellular signalling cascade in THP-1 cells via TLR2, TLR4, or a combination of both receptors [32]. GroEL in all bacterial species has homologous sequences but is constitutively expressed at a low level under physiological conditions [33]. Heat shock proteins, including GroEL, are highly conserved proteins with important biological functions in protein biogenesis. GroEL plays an important role in intracellular protein folding in bacteria. Therefore, GroEL is a key protein involved in intracellular biological functions, protein folding, stress responses, and extracellular immunomodulatory effects. However, secreted GroEL-dependent immunomodulatory effects were highly associated with sporulation and linked with specific characteristic features of the strain in the present study. Therefore, fermentation conditions should be controlled to produce a higher amount of GroEL.

**Figure 9:**
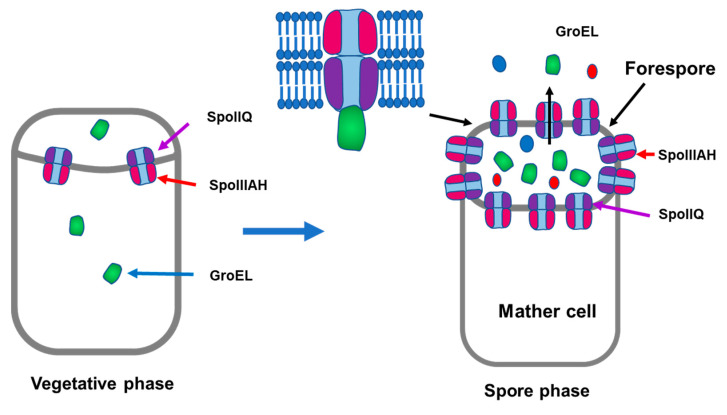
Predicted mechanism of GroEL secretion during *B. subtilis* strain 1 sporulation. Schematic representation of the hybrid transport system during endospore formation. SpoIIQ and SpoIIIAH are coloured purple and red, respectively. The predicted core components of transporter formed with SpoIIIAH and SpoIIQ through the forespore membrane.

Several studies have reported the secretion of various intracellular proteins without signal sequences in *Bacillus* species [34]. Comparative secretome analysis of four *Bacillus* species revealed the interactions of glycolytic enzymes enolase, GroEL, and flagellin protein with host bacterial cells [30]. Several cytoplasmic proteins, including GroEL, DnaK, and enolase, were detected in large amounts during the late stationary phase, suggesting that secretion by *B. subtilis* is not unique to specific strains [30]. In contrast, in this study, GroEL secretion was higher in strain 1, and comparative RNA-seq analysis revealed upregulation of various genes involved in both chaperones, and sporulation was higher in strain 1 than in strain 15 (Figure 7). The relationship between chaperones and sporulation is not clear; however, GroEL secretion in the culture supernatant was higher when strain 1 was cultured in DSM, which was developed for bacterial sporulation [23]. More than 90% of strain 1 cells sporulated in DSM medium. Interestingly, the proteins released from the Natto product prepared with strain 1 showed higher amounts of GroEL than those prepared in DSM (Figure 8B). These findings suggest that GroEL secretion is strongly associated with sporulation. Among the sporulation-related genes, *SpoII* and SpoIIIAH were significantly upregulated in strain 1 (available by request). Recently, a novel transport system involving *SpoIIQ* and SpoIIIAH was proposed to move substrates across the two membranes of developing *B. subtilis* endospores [35,36]. The development of endospores by *B. subtilis* involves the differentiation of the forespore into endospores and mother cells. SpoIIQ produced in the forespore and SpoIIIAH produced in the mother cell interact through two membranes to connect the forespore and mother cell, forming the core components of a channel or transporter through which the mother cell nurtures forespore development [37]. There are no reports on the interaction of GroEL with SpoIIQ or SpoIIIAH; however, a nonspecific secretion system may be involved in GroEL during the sporulation period, as illustrated in Figure 9.

The low risk of breast cancer in Japan and other Asian countries can be explained by a higher intake of fermented soybean products and soybeans [28]. In women, a significant inverse association between fermented soy product intake and cardiovascular disease (CVD) risk was observed in a large cohort study. Another large cohort study in Japan with over 14.8 years of follow-up involving 13,303 deaths revealed that Natto intake showed significant and inverse associations with total cardiovascular disease-related mortality in both sexes [38]. Intake of fermented soybean products such as natto is also inversely associated with the risk of CVD in women [39]. The average consumption of natto products in Japan is approximately 5% and natto produced with 1 strain contained 3.5 × 10^10^ spore-forming cells. The IL-10-inducing activity of strain 1 was higher in THP-1 DC than in *L. acidophilus* JCM 1132. To date, there are no Natto products in the Japanese market that claim the health benefits of boosted immune function. Considering the number of bacterial cells in one Natto product, which is comparable to 100 g of yoghurt-like product containing LAB cells, Natto has the potential to be applied as a functional product in Japan.

## 5. Conclusions

*B. subtilis* natto strain 1, with the highest inducing activity of anti-inflammatory IL-10 and pro-inflammatory IL-12 in THP-1 DC, was selected from 23 *B. subtilis* natto strains isolated from Natto products in Japan to elucidate their immunomodulatory components. The active component was purified from strain 1 culture medium and identified as GroEL, a chaperone protein, which was significantly inhibited by the anti-GroEL antibody. GroEL can interact with the TLR-2 receptor and activate transcriptional regulators, JUN as well as NF-kB, following various host immune responses. GroEL secretion is linked to sporulation and related gene expression. Natto products with a high amount of GroEL have great potential to exert an immunomodulatory effect.

## Figures and Tables

**Table 1:** *Bacillus subtilis* (natto) isolated from various Natto products in Japan.

No	Identified Bacterial Strain	Products	Company	Region
1	*Bacillus subtilis* (natto) 1	Kinnotubu natto	Mizkan	Aichi
2	*Bacillus subtilis* (natto) 2	Okame natto	Takanofoods	Ibaraki
3	*Bacillus subtilis* (natto) 3	Kezurikobushi natto	Yamada Foods	Akita
4	*Bacillus subtilis* (natto) 4	Kiriboshi Natto	NUKAGA SYOJI	Ibaraki
5	*Bacillus subtilis* (natto) 5	Natto-jiman	Hoya-Natto	Tokyo
6	*Bacillus subtilis* (natto)(Rough) 6	Sugoi S-903	Takanofoods	Ibaraki
7	*Bacillus subtilis* (natto)(Smooth) 7			
8	*Bacillus subtilis* (natto) 8	Tezukuri natto	Shika-ya	Kagoshima
9	*Bacillus subtilis* (natto) 9	Hokkaido Kotubu natto	Kajinoya	Kanagawa
10	*Bacillus subtilis* (natto) 10	Yukihomare	Osato	Ibaraki
11	*Bacillus subtilis* (natto) 11	Kawaguchi Natto	Kawaguchi Natto	Miyagi
12	*Bacillus subtilis* (natto) 12	Hamanatto	Suzukishoji	Shizuoka
13	*Bacillus subtilis* (natto) 13	Tukuba-natto	Tukuba-natto	Ibaraki
14	*Bacillus subtilis* (natto) 14	Ohisama natto	Ibaraki	Ibaraki
15	*Bacillus subtilis* (natto) 15	Tengu natto	Ibaraki	Ibaraki
16	*Bacillus subtilis* (natto) 16	Ibaraki hoshi natto	Ibaraki	Ibaraki
17	*Bacillus subtilis* (natto) 17	Namahoshi natto	Ibaraki	Ibaraki
18	*Bacillus subtilis* (natto) 18	Korumame natto	Musouan	Kumamoto
19	*Bacillus subtilis* (natto) 19	Hikiwari natto	Kamakurayama-Noroshokuhinn	Kanagawa
20	*Bacillus subtilis* (natto) 20	Natto-moto	Takahashiyuzo-Kenkyusho	Yamagata
21	*Bacillus subtilis* (natto) 21	Miyagino natto	Miyagino	Miyagi
22	*Bacillus subtilis* (natto) 22	Funmatsu Natto-kin	Naruse Hakkokagaku Kenkyusho	Tokyo
23	*Bacillus subtilis* (natto) 23	Fukuokajiman	Yoshino Shoten	Fukuoka

## Data Availability

The data presented in this study are available by request to corresponding author.

## Supplementary Materials

The following supporting information can be downloaded at: https://www.mdpi.com/article/10.3390/microorganisms11051281/s1, Figure S1: Network analysis of the upregulated genes in THP-1 cells by GroEL treatment; Figure S2: Network analysis of the downregulated genes in THP-1 cells by the GroEL treatment; Figure S3: Network analysis for the upregulated genes in *B. subtilis* natto #1 compared to *B. subtilis* natto #15; Table S1: Upregulated genes in THP-1 DC after GroEL treatment for 4 h; Table S2: Downregulated genes in THP-1 DC after GroEL treatment for 4 h; Table S3: Clusters for the upregulated genes in THP-1 DC; Table S4: Genes showed higher expression in *B. subtilis* #1 than those in #15; Table S5: Clusters for the upregulated genes in *B. subtilis* #1 compared to #15.
